# Health Service Protection vis-à-vis the Detection of Psychosocial Risks of Suicide during the Years 2019–2021

**DOI:** 10.3390/healthcare11101505

**Published:** 2023-05-22

**Authors:** Ismael Puig-Amores, Isabel Cuadrado-Gordillo, Guadalupe Martín-Mora-Parra

**Affiliations:** Department of Psychology and Anthropology, Faculty of Education and Psychology, University of Extremadura, 06071 Badajoz, Spain; ipuigamores@unex.es (I.P.-A.); guadammp@gmail.com (G.M.-M.-P.)

**Keywords:** mental health, primary care, suicide, prevention

## Abstract

Health services are especially relevant in suicide prevention and intervention, representing a favourable environment in which to implement specific strategies to detect and address suicidal behaviours. Indeed, a significant proportion of people who die by suicide (DBS) present at primary care and mental health services during the last year, month, or even days before committing suicide. The objective of this descriptive and cross-sectional study of all registered cases of death by suicide (*N* = 265) in Extremadura (Spain) was to determine which of those people who died by suicide had mental health problems (MHP) and what type of assistance they had requested. Diagnoses, previous suicide attempts, type of health service, and last visit before death were explored with univariate analyses and logistic regressions. The proportion of people without MHP was found to be high, and these people had hardly visited the health services at all in their last year. People with MHP, between the ages of 40 and 69, and with previous suicide attempts were more likely to have visited the mental health service in the three months prior to their death. It is, thus, necessary to provide health professionals with tools and training in the prevention of and approach to suicide. Efforts must be directed towards effectively assessing mental health and the risk of suicide since a large proportion of people who die by suicide may go unnoticed.

## 1. Introduction

Suicide is a grave global public health problem that affects around 800,000 people every year [1]. While the global rate has decreased slightly since the year 2000 [2], in Spain, and specifically in the region of Extremadura, the rate has remained relatively stable in the last ten years [3,4].

The multifactorial nature of suicide makes it a complex phenomenon, and its prevention requires comprehensive and multisectoral strategies [1]. As one of these sectors, the healthcare system is particularly relevant for suicide prevention and intervention and represents a favourable environment in which to implement specific strategies for the detection and approach to suicidal behaviours. Indeed, a significant proportion of people who die by suicide present at the different health centres (primary care and mental health) during the last year, months, or even days before committing suicide [5,6,7,8]. Most of them suffer from mental health problems, which include mental disorders and psychosocial disabilities as well as other mental states associated with significant distress, impairment in functioning, or risk of self-harm [9]. Additionally, suicide attempts are more frequent among people who suffer or have suffered from psychological and emotional disorders [8,10,11,12,13]. In this sense, some studies [5] estimate that a major proportion of these deaths are associated with mental health problems, although the actual figure reported varies according to different authors [14]. Nevertheless, suicidality can occur in the absence of any identifiable risk factor (for example, psychiatric history or prior suicide attempt) [14,15,16,17] so that these predispositions may sometimes go unnoticed in health services since the person involved is considered to be low risk [18]. Therefore, is important to highlight that many people with psychological problems have never had contact with specialized services, and their treatment and follow-up is carried out in the context of primary care [5,6,19]; for this reason, this service is essential for the prevention of suicide. Indeed, some studies [20] have noted that people who commit suicide have frequently visited this service. Likewise, mental health service is an environment prepared to detect and prevent suicidal behaviours since it is common for people with mental health problems and a history of suicide attempts to be monitored with this service [21,22].

With respect to the current state of suicide prevention, there is extensive literature on the effectiveness of its early detection [23,24]. This interest arises from the need to determine the specific characteristics of suicide risk in people with or without psychological problems who receive health care [25,26,27]. Some studies [27,28] show that the identification of suicide risk needs to evolve towards more precise detection strategies than those based on the general factors traditionally associated with suicidal behaviour (mental health problems, chronic illness, loneliness, hopelessness, etc.). For this reason, work is currently underway to construct predictive models using algorithms based on clinical information [29,30,31].

However, although suicide prediction models generate precise classifications, their effectiveness in predicting a future event remains low [32]. In addition, to make the implementation of this type of suicide risk detection system effective, the data the system requires to carry out the predictions need continuous updating, and this methodology requires highly sophisticated computing capacity [33]. Consequently, future practice points towards clinical risk assessment carried out by health professionals with the support of statistical information from the health system [7,16,29,34]. In this sense, traditional research on suicide risk factors and the use of tools to detect them, although with limitations, is still an essential component of the set of strategies that can help identify individuals at risk [23,35,36,37,38].

The present work follows the recommendations of the WHO [1] on the need to continue investigating the risk factors associated with suicidal behaviour in certain geographical areas and in specific populations at risk. In this context, the objectives of this study were to determine (i) which people had mental health problems among those who died by suicide in the region of Extremadura (Spain) during the years 2019–2021 and (ii) what type of assistance was demanded (primary care or mental health).

## 2. Materials and Methods

### 2.1. Study Design

**Figure 1:**
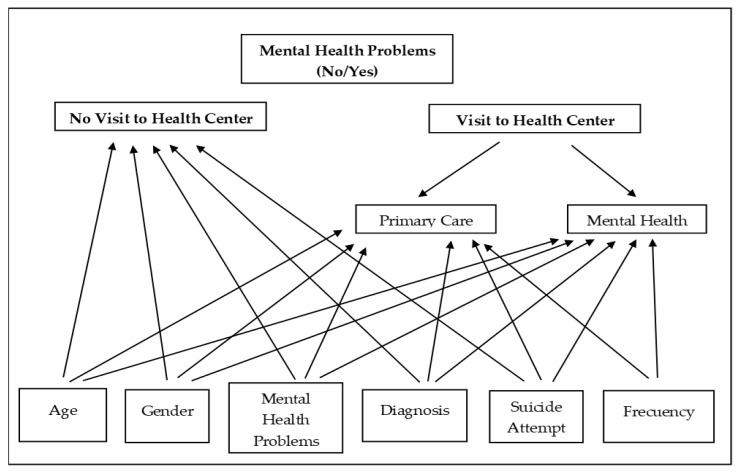
Study design.

This study was descriptive and cross-sectional. The sample was divided into two groups (mental health problems yes/no). In turn, these two groups were subdivided into four groups: visit to health services (yes/no) and type of health service (mental health/primary care). The demographic and clinical study variables were the year of the case, sex, age, diagnosis, suicide attempts, and visits to health centres (type of service and last visit). The design compares the four groups to explore the differential characteristics between the people who presented with problems of mental health and those who did not and then to explore whether there were differential characteristics between the people who go to primary care or mental health. Specifically, the interest of this study was to analyse the study variables in the group of people with mental health problems who attend health services since they are the group with the greatest possibility of being detected by health professionals. Figure 1 shows the final study design.

### 2.2. Participants

The study population (*N* = 265; male = 83%; female = 17%) consisted of all registered cases of death by suicide in Extremadura (Spain) from 1 January 2019 to 31 December 2021. The ages of the participants were from 19 to 94 years old (M = 56.52; SD = 17.963).

### 2.3. Data Acquisition and Procedure

The data were extracted from the records of deaths by suicide (DBS) of the Provincial Institutes of Legal Medicine and Forensic Sciences of Cáceres and Badajoz (IMLyCF, the two provinces that comprise the Extremadura Region), with the approval of the teaching commission of these centres and the authorization of Spain’s Ministry of Justice, both subject to organic law 3/2018 of 5 December, protection of personal data, and guarantee of digital rights. Additional complementary information provided by the Comandancia of Extremadura’s Guardia Civil was incorporated. The complementary information gathered is the demographic data (sex and age) was missing in the IMLyCF dossiers.

The records for the years 2019–2021 were reviewed, and the DBS that occurred in that period were collected, incorporating the variables of study (Figure 1). All variables were coded dichotomously (yes/no) except year and age, which were classified into 3 groups each (2019; 2020; 2021) and (<40; 40–69; >69) respectively.

### 2.4. Statistical Analysis

The statistical analysis was performed using the Statistical Package for the Social Sciences (SPSS v.26; IBM Corp. Armonk, NY, USA). Firstly, a descriptive analysis was performed to determine the distribution of the independent variables (demographic and clinical) in the study population. Next, the population was divided into two groups: “persons with mental health problems” and “persons without mental health problems”. Subsequently, exploratory analyses were carried out in both groups searching for potential associations between the independent variables and the dependent variable “Visits to Health Centres” (yes/no). Secondly, the relationships between the dependent variable “type of service” (mental health centre or primary care centre) and the independent variables were explored using univariate analyses. As a control, the possible interactions between all these variables were checked. These analyses were performed using Pearson’s chi-squared univariate test by layers and Fisher’s exact test for dichotomous and polytomous variables.

Finally, the possible relationships between the demographic and clinical variables and the type of health service used were explored applying binary backwards stepwise (conditional) logistic regression. These regression analyses included the set of variables that were statistically significant in the univariate analyses as well as the significant interactions between them. The odds ratios (OR) resulting from these tests indicate a greater likelihood of an event occurring when OR > 1 and the lower bound of the confidence interval (CI) is greater than 1 and a lower likelihood of occurrence when OR < 1 and statistical significance when the CI upper bound does not exceed 1.

## 3. Results

### 3.1. Analysis of the Demographic and Clinical Characteristics

Table 1 presents the results of the descriptive analysis of the 265 cases covered in this study. One observes in the table that the DBS were distributed uniformly with respect to the year of the case. Based on sex, most of the cases were men (X^2^ = 115.566; *p* < 0.001), of whom a large percentage (55.5%) were in the 40 to 69 years age group (X^2^ = 63.530; *p* < 0.001). Slightly more than half suffered from mental health problems (55.1%; ns). Previous suicide attempts were a relatively infrequent phenomenon (17.4%; *p* < 0.001).

With regard to mental health problems, mood disorder was present in 38.9% of the cases, being the most frequent of the diagnoses (X^2^ = 218.226; *p* < 0.001). Substance abuse and other disorders (psychotic and personality) were less frequent. Only 6.1% of the people had a chronic illness. With regard to visits to health services, only slightly more than half of the people (57.36%; *p* < 0.001) had made one or more during the last year (35.8%; *p* < 0.001) or the last three months (21.8%; *p* < 0.001) before committing suicide.

### 3.2. Descriptive Analysis of the DBS Group of Persons without Mental Health Problems

This section presents the results of the descriptive analysis of the DBS group of persons with no known psychiatric history (Table 2). This group represents 44.9% of the DBS cases and is distributed uniformly with respect to the year of the case. As can be seen, only 8.1% of the persons without mental health problems were attended to in a health centre (X^2^ = 134.247; *p* < 0.001).

With regard to the demographic variables, men committed suicide to a greater extent than women (X^2^ = 52.445; *p* < 0.001), with the largest age group being that of 40 to 69 years, being significantly older than the mean age of the rest (X^2^ = 30.672; *p* < 0.001). However, no significant association was found between having visited health services and any of the demographic and clinical variables analysed, with only one DBS case recorded in this group of persons (Table 2).

### 3.3. Descriptive Analysis of the DBS Group of Persons with Mental Health Problems

This section presents the results of the descriptive analysis of the group of persons with mental health problems. The distribution for the variables year of the case, age, and sex are similar to those of the group of persons without a history of psychopathology (Table 2 and Table 3). Nearly 5 times more men committed suicide than women during the period studied (Χ^2^ = 63.123; *p* < 0.001), there were more cases in the 40 to 69 years age group (Χ^2^ = 34.78; *p* < 0.001), and there were no differences with year of the case.

With respect to visits to health centres, 97.9% of persons with a psychiatric history attended one of the health services (primary care or mental health) (X^2^ = 218.972; *p* < 0.001), 30.1% of whom had made a previous suicide attempt.

Comorbid mental health problems were found among those who suffered from a chronic illness (X^2^ = 12.659 (<0.001); *p* < 0.001). Additionally, all these cases had consulted a health service in the last three months (X^2^ = 55.200 *p* < 0.001) or in the last year before committing suicide (X^2^ = 108.296; *p* < 0.001).

The results presented up to now show that persons with no known psychopathological history attended health services hardly at all. This led us to consider that a comparative analysis based on the possible differences between the groups (with or without mental health problems) would not provide additional information. To this end, the following subsection presents a more exhaustive analysis considering just the group of persons with mental health problems and their relationship with the different health services (primary care and mental health).

### 3.4. Univariate Analysis of the Cases with Mental Health Problems

Table 4 lists the results of the univariate analysis of the group of persons with mental health problems and their relationship with the different health services. The Pearson chi-squared test showed that, in this group of persons, the variables year of the case, sex, age, diagnosis, previous suicide attempts, and last visit were significantly correlated with the type of service. Thus, only 3 (2.1%) people had not attended health services in the previous year. Of the 143 cases that did maintain contact with health services, 67.3% did so with primary care and the rest (32.7%) with mental health. This result indicates that persons with mental health problems visited the primary care service to a greater extent than the mental health services (X^2^ = 10.636; *p* = 0.001).

With regard to the demographic variables, a significant relationship was found between the year of the case and visits to health centres. This finding indicated a greater number of visits during the year 2020 compared to the years 2019 and 2021 (*p <* 0.05). Likewise, a statistically significant relationship was found between the sex variable and the type of service that men and women attended (*p* < 0.05). Of the women, 54.2% attended specialized mental health care services compared with 32.8% of the men, with the latter also being the cases who, to a greater extent, visited primary care services (*p* < 0.001). By age, persons between the ages of 40 and 69 visited specialized mental health care services more frequently than the rest of the age groups (*p* < 0.005), while persons over 70 years of age attended primary care services more frequently (*p* < 0.05) (OR = 0.450; CI = 0.204–0.991).

With respect to the assessment of mental health, problems related to mood occurred in a greater proportion than the rest (*p* < 0.001). However, only substance abuse and such diagnoses as psychosis, dementia, and personality disorder were significantly related to the type of health service (*p* < 0.01; *p* < 0.001). Additionally, it was found that none of the persons diagnosed with substance abuse visited specialized mental health services, but all were seen in primary care (*p* < 0.01). In contrast, persons with psychosis, dementia, and personality disorders mostly attended mental health services (*p* < 0.001).

Previous suicide attempts were also significantly correlated with the type of health service (*p* < 0.005). It is noteworthy that almost half of the persons with previous attempts were treated in primary care (OR = 1.578; CI = 1.116–2.231) and that almost half of the persons who attended specialized services had attempted suicide at least once before dying by suicide (OR = 3.043; CI = 1.456–6.360).

Finally, the analysis showed that the last visit was significantly correlated with the type of service attended (*p* < 0.001). Thus, approximately 60% of the persons who visited mental health services did so in the last few months before DBS. This indicates that the visits in the last 3 months are linked to mental health services, while the majority of persons who attended primary care did so in the last year before committing suicide (*p* < 0.001).

### 3.5. Logistic Regression Analysis

Two binary logistic regressions were performed in order to explore the way in which the independent variables (year of case, sex, age, suicidal attempts, diagnosis, and last visit) were related to each of the health services visited. Table 5 and Table 6 show the results of these analyses.

In the first, multivariate logistic regression was applied to test sex, age, previous suicide attempts, and diagnosis. As a control measure, the significant interactions between these variables were included. The test of the regression model’s fit was valid (*p* < 0.001), and Nagelkerke’s R^2^ was moderate (0.362). As one observes in Table 5, there were significant associations with the 40 to 69 years age group (*p* = 0.016), previous suicide attempts (*p* = 0.027), diagnosis of psychosis (*p* = 0.001), and visiting health services in the previous three months (*p* < 0.001) using specialized mental health services. These results show that people between 40 and 69 years old and those who had attempted suicide were almost three times more likely to seek mental health services. In this sense, people diagnosed with psychosis were almost 14 times more likely to use this health service.

We included in a second logistic regression the year of the case variable as well as all the interactions between the variables that were significant in the univariate analysis (Table 6). The equation of the regression model was valid (*p* < 0.001), and Nagelkerke’s R^2^ was 0.395. A Hosmer–Lemeshow test showed the model’s fit to be good (*p* = 0.499). The final regression model showed that the year of the case (year 2020; *p* = 0.022), visits in the last 3 months (*p* < 0.001), suicide attempts in persons aged between 40 and 69 years (*p* < 0.001), and the diagnosis of psychosis (*p* = 0.001) were associated with mental health services.

## 4. Discussion

This study replicates some universal results on the demographic characteristics associated with suicides, such as the most prevalent age (40–60 years old) (OMS, 2014) [1] and the great difference in terms of sex, finding up to three times more deaths among men than women (OMS, 2014) [1]. The difference in terms of sex has traditionally been explained by the use of more lethal methods by men [1]. On the other hand, regarding the variable “year of the case”, in line with several studies (Puig-Amores et al., 2021) [4], during 2020 (COVID-19 pandemic year), there was not a great difference by suicide compared to the previous year and the following year. However, our results indicate that during 2020, people who committed suicide made more use of mental health services compared with the previous years. This result could be related with the increase of mental health problems observed after the period of confinement. The duration of the confinement, the fear of getting ill, the frustration caused by being unable to carry out daily activities such as leaving the house, the loss of freedom, the separation from one’s environment, and uncertainty were some of the psychosocial stressors associated with the health situation that were potential generators of negative psychological effects during the pandemic [39].

In line with recent research [16], most of the people in our study population who died by suicide had mental illness, although many of them were neither diagnosed nor treated with specialized services [5,6]. Bearing in mind that suffering from psychological problems does not necessarily imply suicidal behaviour, the risk is often underestimated and even more so when there have been no previous suicide attempts [18]. Something similar could have occurred in this study population. Indeed, a history of suicide attempts was relatively infrequent (17.4%), a figure close to that obtained in another recent study [17] which examined DBS that occurred between 2005 and 2013 and which showed that 79% of these persons consummated the act on the first attempt.

Almost half of the persons who died by suicide had no known psychiatric history, as has been seen in previous studies [17]. Therefore, these persons had maintained hardly any relationship with health services during the last year before dying, as is quite frequent in this population [8]. However, it seems more than likely that those with no diagnosis who died by suicide suffered some subclinical mental health characteristics, a fact suggested by previous research such as that carried out by Joiner et al. [40]. This reality implies the existence of a group of people at risk of suicide who are difficult to identify and, consequently, quite difficult for their suicide to be prevented from the health field. Likewise, in line with some previous studies [5,6], our results indicate that most of the DBS cases in Extremadura in the period 2019–2021 had attended primary care services to a greater extent in the months before dying.

The lack of specialized mental health care among the persons who suffered psychological problems associated with some type of chronic illness suggests that in some cases, as observed in this sample, the primary care physician takes on the supervision of many of their patients in matters of mental health (63.7% in this study). This fact points to the need to review the primary care protocols regarding the approach to patients with chronic illnesses and comorbid mental health problems. In this sense, the suicide prevention plan of the study region [41] insists on training health professionals in the detection and intervention of suicidal behaviours as an effective preventive strategy. This same fact has been corroborated by various authors in diverse studies [42,43,44,45,46]. Nonetheless, these preventive measures would not be enough, with an additional review of guidelines such as consultation time being necessary. Previous studies [47] have shown that primary care physicians have barely 6 min per patient, which is clearly an insufficient amount of time to make a good diagnosis in cases such as these. Evidence-based best practices address the fluctuating nature of suicide risk, noting that ongoing risk assessment, direct intervention, and long-term follow-up are required [48].

In this sense, our study provides information of interest for the work of suicide prevention in health services, pointing out some clinical characteristics present in people who died by suicide in the last three years and opening up a route of observation to improve the estimation of the risk of suicide in those people who go to health services in search of help. Additionally, the strength of this study lies in providing a warning about the need to increase resources to improve the effectiveness of professionals in clinical practice, with this being especially relevant in primary care, since a large part of people who commit suicide each year pass through this service in the last year and in the three months prior to committing suicide [46].

Taking the above into account, although there has been progress and an effort in relation to public policies aimed at confronting and preventing suicide [41], it continues to be a great challenge for health professionals to characterize and identify people at an increased risk of attempting and completing suicide. Faced with this fact, it becomes important to know the characteristics of the individuals who attempt or commit suicide so that new strategies can be generated to avoid that consummation and so that there can be assistance in the treatment of those who attempted suicide. Therefore, an important practical implication of the present study for clinical practice and for the development of suicide prevention policies is precisely the incorporation of these results, which were obtained from the analysis of clinical data, into the prevention protocols of primary care services. With this, it can be expected that routine examinations in primary care will be more effective in detecting risk. Providing that, it should be noted that the evaluation of the suicidal patient must continue to be the responsibility of an expert mental health professional who has to combine clinical judgment with knowledge of empirically validated risk and protective factors and weigh these factors in the best way possible [16]. However, as has been shown in the present research and in previous studies [47,49], this expert’s work is not necessarily limited to specialized mental health services, since the role of primary care is essential in the screening and follow-up of patients. Thus, a greater attention to specialized training for the detection of suicide would be another key factor in effective prevention, including all professionals in contact with the population at risk.

## 5. Conclusions

This research confirms that in the study region there are a large number of people with no psychiatric history who die by suicide each year and who, furthermore, make very little use of health services during the previous year or months. This reality implies that there is a group of persons who are difficult to identify in health services, and, consequently, this fact confirms that it is a multi-causal phenomenon that must be addressed from the different sectors involved if deaths by suicide are to be reduced. Likewise, this study reveals that people with mental health problems go to health services, especially primary care, to a greater extent than people without this type of problem. At the same time, it was noted that there are some specific characteristics (diagnosis, age group, previous attempts, and when the last visit was made) which are associated with people who go to specialized mental health services. Additionally, among people with mental health problems, those who had made a previous suicide attempt frequented health services to a greater extent than those who had not made any suicide attempt. This fact indicates that people with mental health problems and, to a greater extent, those who had attempted suicide before consummating the act went to health services in the last year and in the last three months. Consequently, we can affirm that this group of people were being monitored to a greater or lesser extent so that, a priori, there was an opportunity to identify the risk of suicide in these cases. In this sense, the greatest strength of this study lies in revealing some of the factors that should alert mental health specialists, understanding, in addition, that the risk of suicide should in no case be underestimated. In conclusion, there are opportunities for suicide prevention in medical settings, and efforts should be directed towards better identification of mental illness and suicidal ideation.

## 6. Limitations

Our research has some limitations to take into account. While the exploratory, observational, and retrospective design has generated valuable information for clinical practice, it is important to point out that more detailed data collection by professionals would help to complete the findings. While it is true that the protocols for writing up reports give clear guidelines on how they should be completed, making an additional effort to standardize the said collection would provide a greater number of variables. In this sense, future lines of research could explore the effect that variables related to vulnerability factors (marital status, cohabitation, previous life events, etc.) have on suicide. In addition, following the recommendations of the WHO [1] and given the main interest of this work, this research was carried out in a specific geographical area and on a specific population at risk, which provided valuable and useful results and conclusions at the contextual level. Nevertheless, this study has not expanded to other regions with potentially different profiles and characteristics.

## Figures and Tables

**Table 1:** Analysis of the demographic and clinical variables of DBS.

	Deaths by Suicide (N 265)	
	*N* (%)	X^2^ (*p*-Value)
**Health Centre visits**		
No	113 (42.64)	(n.s.)
Yes	152 (57.36)	
**Year of case**		
2019	90 (34)	
2020	93 (35)	(n.s.)
2021	82 (31)	
**Sex**		
Male	220 (83)	115.566 (*)
Female	45 (16)	
**Age**		
<40 years	44 (16.6)	
40–69 years	147 (55.5)	63.540 (*)
>70 years	74 (27.9)	
**Mental Health Problems**		
No	119 (44.9)	(n.s.)
Yes	146 (55.1)	
**Diagnosis**		
Mood disorders	103 (70.55)	218.226 (*)
Substance abuse	10 (6.84)	
Others (psychotics, dementia, personality)	17 (11.64)	
**Chronic Illness**		
No	249 (93.9)	204.864 (*)
Yes	16 (6.1)	
**Suicidal Attempts**		
No	219 (82.6)	112.940 (*)
Yes	46 (17.4)	
**Visit** **three months prior**		
No	207 (78.2)	83.777 (*)
Yes	58 (21.8)	
**Visit last year prior**		
No	171 (64.2)	22.374 (*)
Yes	94 (35.8)	

(*) *p* < 0.001 = significant. (n.s.) = non-significant.

**Table 2:** Descriptive analysis of the DBS cases without mental health problems.

	Deaths by Suicide without Mental Health Problems			
	***N* (%)** 119 (44.9)			
	**Health Centre Visits**			
	**No**	**Yes**	**Total**	**Chi Square (*p*-Value)**
	***N* (%)**	***N* (%)**	***N* (%)**	
**Health Centre visits**	110 (91.9)	9(8.1)	119	134.274 (*)
**Year of case**				
2019	35 (89.7)	4 (10.3)	39 (43.3)	
2020	40 (9.3)	3 (90.7)	43 (46.2)	n.s.
2021	35 (94.6)	2 (5.4)	37 (45.1)	
**Sex**				
Male	91 (75)	8 (25)	99 (37.4) *	n.s.
Female	19 (95)	1 (5)	20 (44.4)	
**Age**				
<40 years	18 (78.3)	5 (21.7)	23 (52.3)	
40–69 years	64 (94.1)	4 (5.9)	68 (46.3) *	n.s.
>70 years	28 (100)	-	28 (51.1)	
**Chronic Illness**	-	-	-	-
**Suicidal Attempts**	1 (100)	-	1 (2.2)	-
**Visit** **three months prior**	-	3 (100)	3 (4.8)	-
**Visit last year prior**	-	6 (100)	6 (6.3)	-

(*) *p* < 0.001 = significant. (n.s.) = non-significant.

**Table 3:** Descriptive analysis of the DBS cases with mental health problems.

	Deaths by Suicide with Mental Health Problems			
	***N* (%)** 146 (55.01)			
	**Visit to Health Centre**			
	**No**	**Yes**	**Total**	**Chi Square (*p*-Value)**
	***N* (%)**	***N* (%)**	***N* (%)**	
**Health Centre visits**	3 (2.1)	143 (97.9)	146	218.972 (<0.001) (*)
**Year of case**				
2019	1 (2)	50 (98)	51 (56.7)	
2020	-	50 (100)	50 (53.8)	(n.s.)
2021	2 (4.4)	43 (95.6)	45 (54.9)	
**Sex**				
Male	2 (.99)	119 (99.1)	121 (62.6) *	(n.s.)
Female	1 (4)	24 (96)	25 (55.6)	
**Age**				
<40 years	-	21 (100)	21 (47.7)	(n.s.)
40–69 years	2 (2.5)	77 (97.5)	79 (53.7) *	(n.s.)
>70 years	1 (2.2)	45 (97.8)	46 (48.9)	(n.s.)
**Chronic Illness**	-	16 (100)	16	12.659 (<0.001) (*)
**Suicidal Attempts**	1 (2.2)	44 (97.8)	45 (97.8)	33.372 (<0.001) (*)
**Visit** **three months prior**	-	55 (100)	58 (95.2)	55.200 (<0.001) (*)
**Visit last year prior**	-	88 (100)	88 (93.7)	108.296 (<0.001) (*)

(*) *p* < 0.001 = significant. (n.s.) = non-significant.

**Table 4:** Univariate analysis of the group of persons with mental health problems according to type of health service.

Health Centre (Type)				
		Primary Care	Mental Health	
		*N* (%)	*N* (%)	Chi Square (*p*-Value) Odd Ratio (C.I.: 95%)
Variable		91 (63.7)	52 (36.3)	
**Health Care**		91	52	10.636; *p* = 0.001 *
**Year of case**				
2019	No	57 (61.3)	36 (38.7)	-
	Yes	34 (68)	16 (32)	-
2020	No	66 (71) *	27 (29)	6.178 (0.013) *
	Yes	25 (50)	25 (50)	2.444 (1.199–4.985) *
2021	No	59 (59)	41 (41)	-
	Yes	32 (74.4)	11 (25.6)	-
**Sex**				
Male		80 (67.2) *	39 (43.3) *	3.950 (0.047) *
Female		11 (45.8)	13 (54.2)	2.424 (0.996–5.900)
**Age**				
<40 years	No	74 (60.7)	48 (39.3)	-
	Yes	17 (81) *	4 (19)	-
40–69 years	No	51 (77.3)	15 (22,7)	9.849 (0.002) *
	Yes	40 (51.9)	37 (48.1) *	3.145 (1.517–6.519) *
>70 years	No	57 (58.2)	41 (41.8)	4.031 (0.045) *
	Yes	34 (75.6)	11 (24.4)	0.450 (0.204–0.991) *
**Mental Health Problems**		91 (63.7)	52 (36.3)	20.664 (0.000) *
**Diagnosis**				
Mood Disorders	No	28 (63.6)	16 (36.4)	-
	Yes	63 (63.6) *	36 (36.4) *	19.973 (<0.001) *
Substance abuse	No	81	52	6.144a (0.009) *
	Yes	10	-	
Others (psychotics, dementia, personality)	No	88 (68.8)	40 (31.3)	13.789 (0.000) *
	Yes	3 (9.5)	12 (23.1)	8.800 (2.353–32.916) *
Chronic Illness	No	79 (62.2)	48 (37.8)	-
	Yes	12 (75)	4 (25)	-
**Suicidal attempts**				
	No	71 (71.7) *	28 (28.3)	9.079 (.003) *
	Yes	20 (45.5)	24 (54.5)	3.043 (1.456–6.360) *
**Health centre**				
Visit three months prior	Yes	25 (45.5)	30 (54.5) *	12.768 (0.000) *
Visit last year prior	Yes	66 (75) *	22 (25)	3.600 (0.757–7.376) *

(*) *p* = significant. (n.s.) = non-significant.

**Table 5:** Results of the multivariate analysis (1).

	B	*p*-Value	O.R.	C.I. 95%	
**40 to 69 years**	1.022	0.016	2.750	1.212	6.376
**Suicidal Attempts**	0.989	0.027	2.689	1.121	6.451
**Visit** **three months prior**	1.749	0.000	5.752	2.440	13.556
**Psychosis**	2.626	0.001	13.816	3.044	62.701

**Table 6:** Results of the multivariate analysis (2).

Variables	B	*p*-Value	O.R.	C.I. 95%	B
**Year 2020**	0.992	0.022	2.696	1.151	6.321
**Visit** **three months prior**	1.928	0.000	6.878	2.795	16.928
**Suicidal attempts by 40 to 60 years**	2.490	0.000	6.692	2.256	1.848
**Psychosis**	1.901	0.001	12.071	2.600	56.041

## Data Availability

Data is contained within the article.
